# Gamma-hydroxybutyrate to promote slow-wave sleep in major depressive disorder: a randomized crossover trial

**DOI:** 10.1038/s41386-025-02104-4

**Published:** 2025-04-14

**Authors:** Francesco Bavato, Laura K. Schnider, Dario A. Dornbierer, Julia R. Di Floriano, Benjamin Stucky, Nicole Friedli, Marina Janki, Boris B. Quednow, Hans-Peter Landolt, Oliver G. Bosch, Erich Seifritz

**Affiliations:** 1 Department of Adult Psychiatry and Psychotherapy, University Hospital of Psychiatry Zurich, University of Zurich, Zurich, Switzerland; 2 Institute of Pharmacology and Toxicology, University of Zurich, Zurich, Switzerland; 3 Neuroscience Center Zurich, University of Zurich and Swiss Federal Institute of Technology Zurich, Zurich, Switzerland; 4 Sleep & Health Zurich, University of Zurich, Zurich, Switzerland

**Keywords:** Outcomes research, Drug development

## Abstract

In major depressive disorder (MDD), main clinical features include insomnia and increased daytime sleepiness. However, specific treatment options to promote sleep in MDD are limited. Gamma-hydroxybutyrate (GHB, administered as sodium oxybate) is a GHB/GABA_B_ receptor agonist used clinically in narcolepsy, where it promotes restorative slow-wave sleep (SWS) while reducing next-day sleepiness. Hence, we performed a randomized, placebo- and active comparator-controlled, double-blind, crossover trial to investigate the sleep-promoting properties of GHB in individuals with MDD. Outpatients aged 20–65 years fulfilling the DSM-5 criteria for MDD were enrolled. A single nocturnal dose of GHB (50 mg/kg) was compared with a single evening dose of the clinical competitor trazodone (1.5 mg/kg) and placebo. Of 29 randomized patients, 23 received at least one intervention and were included in the analysis. Primary outcomes were nocturnal slow wave sleep ([SWS] assessed by polysomnography), next-day vigilance (median response time and number of lapses on the psychomotor vigilance test [PVT]), next-day working memory (median speed and accuracy on an N-back task), and next-day plasma brain-derived neurotrophic factor (BDNF) levels. GHB robustly prolonged SWS compared to both trazodone and placebo. GHB also prolonged total sleep time and enhanced sleep efficiency, while reducing sleep stages N1, N2, and wake-after-sleep-onset. While the median response time on the next-day PVT was unaffected, GHB reduced the number of lapses compared to trazodone and placebo. No effects on next-day working memory performance and BDNF levels were observed. No serious adverse events occurred. Overall, a single nocturnal dose of GHB effectively promotes SWS and shows more favorable effects on next-day vigilance than trazodone and placebo. Future studies should investigate GHB in clinical settings, including repeated administration.

## Introduction

Major depressive disorder (MDD) is a serious mental health condition, which is considered the largest single contributor to global disability according to the World Health Organization [1]. Across the clinical presentations of MDD, impairments of sleep are some of the most consistently observed symptoms [2]. Sleep disturbances in MDD include prolonged sleep latency, frequent nocturnal awakenings, non-restorative sleep, and daytime sleepiness [3]. Polysomnographic (PSG) investigations demonstrated that decreased slow-wave-sleep (SWS) and increased rapid-eye-movement (REM) sleep duration are hallmarks of nocturnal sleep alterations in MDD, although large inter-individual variability is observed [4]. Notwithstanding, current pharmacological options to treat sleep in patients with MDD show unsatisfactory outcomes, as most compounds may actually reduce restorative SWS and increase daytime sleepiness [5, 6].

Gamma-hydroxybutyrate (GHB, clinically administered as sodium oxybate) acts as an agonist at GABA_B_ and GHB binding sites, which modulate homeostatic functions such as eating, sexual behavior, and sleep [7, 8]. The latter effect is clinically used in narcolepsy, where GHB strongly enhances nocturnal SWS, while reducing next-day sleepiness and cataplexy [9]. Potential clinical applications of GHB to treat excessive daytime sleepiness have been suggested in different neuropsychiatric disorders including fibromyalgia and Parkinson’s disease [10, 11]. However, to our knowledge, no study has specifically investigated nocturnal GHB administration in MDD.

Based on these considerations, we conducted a randomized, balanced, double-blind, placebo-controlled, crossover trial to investigate the effects of a single nocturnal administration of GHB vs. trazodone vs. placebo in patients with MDD. Our primary objectives were to determine the effect of GHB on SWS, next-day sustained attention, (assessed with the psychomotor vigilance test [PVT]), and next-day working memory (assessed with an N-back task). Plasma brain-derived neurotrophic factor (BDNF) was measured as putative marker of rapid antidepressant effects. Secondary objectives included self-reported sleep quality, sleepiness post-awakening, and next-day affective state. The single nocturnal administration of GHB was selected instead of the classical narcolepsy scheme (two administrations per night with slow titration over weeks) to test the efficacy of single add-on treatments on sleep and next-day mental state. This approach might increase the feasibility of GHB administration in various clinical settings, not requiring titration, prolonged administration, or follow-up monitoring [12]. Trazodone, one of the most frequently prescribed off-label medications for sleep disturbances in MDD, was selected as active comparator because of the SWS-promoting effects and relatively short half-life [13]. We hypothesized that both GHB and trazodone would increase the duration of restorative SWS (hypothesis: GHB>trazodone>placebo). In contrast to trazodone, however, we expected GHB to promote next-day sustained attention and working memory as measured by performance on the PVT and the N-back task (hypothesis: GHB > placebo > trazodone). This project represents the first proof-of-concept study on GHB as a sleep medication in MDD.

## Methods

### Overview

**Fig. 1:**
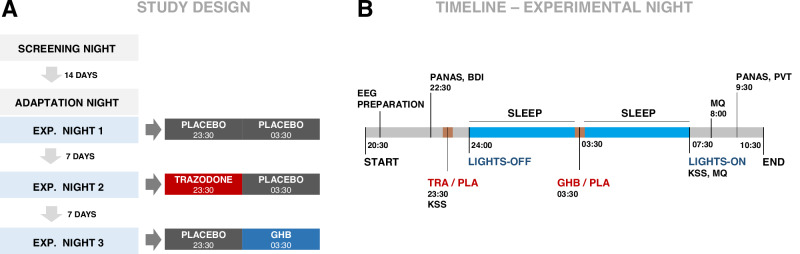
Study design. **A** Study design consisting of one screening night, one adaptation night, and three experimental nights. **B** Timeline of an experimental night. The study medications were administered at two points during the night, with trazodone or solid placebo at 23:30 and GHB or liquid placebo at 03:30. PANAS Positive and Negative Affective Scale, BDI Beck Depression Inventory, MQ Morning Questionnaire, PVT Psychomotor Vigilance Test, KSS Karolinska Sleepiness Scale, EQ Evening Questionnaire.

The study was performed as single-center trial between August 2020 and April 2022 in the sleep laboratory at the Institute of Pharmacology and Toxicology, University of Zurich, Switzerland. Formal approval was obtained by the Swiss Agency for Therapeutic Products (Swissmedic) and the Ethics Committee of the Canton of Zurich (Identifier: 2018-01293). All participants provided written informed consent according to the declaration of Helsinki and received a monetary compensation for their study participation. The study followed a randomized, order-balanced, double-blind, crossover design and tested GHB against the clinical competitor trazodone, and placebo (Fig. 1). The study protocol consisted of five non-consecutive nights: a screening night including a medical visit and all-night PSG examination, an adaptation night to allow for habituation to the laboratory setting, and three experimental nights (GHB, trazodone, and placebo conditions in random order). The three experimental nights were separated by washout phases of 7 days each.

### Participants

Out of 31 patients with MDD initially assessed for eligibility, 29 patients were enrolled into the study and allocated to randomization, 23 received at least one intervention and were included in the analysis, and 22 patients completed the entire study protocol (see “CONSORT flowchart”, Figure S1). A group of healthy volunteers was also studied to investigate the role of pharmacological sleep enhancement on memory functions but was not considered for the present analysis. All participants were assessed by an experienced study physician regarding their general health and psychiatric history. Clinician administered questionnaires included the Montgomery-Åsberg Depression Rating Scale and the Hamilton Depression Rating Scale (HAMD). Self-reported sleep quality was assessed at the screening with the Pittsburgh Sleep Quality Index. At the beginning of each session, self-reported depressive symptoms were assessed with the Beck Depression Inventory. Inclusion criteria were as follows: diagnosis of MDD according to the Diagnostic and Statistical Manual of Mental Disorders (DSM-5); stable antidepressant treatment (e.g., selective serotonin reuptake inhibitors [SSRI] or serotonin–norepinephrine reuptake inhibitors [SNRI]); age between 20 and 65 years; no or low dependence on nicotine according to Fagerström Test for Nicotine Dependence (total score < 3). Exclusion criteria entailed: intake of any sleep-promoting medication, such as benzodiazepines or z-drugs, 3 days before an experimental night or on a regular basis; any axis-I DSM-5 psychiatric disorder other than MDD; neurological disorders or head injury; any clinically relevant medical diseases. Detailed exclusion criteria are reported in the Supplementary Methods.

### Interventions

Study medications were administered at two predefined times during each experimental night (Fig. 1). Liquid GHB or placebo (0.9% natrium chloride solution) were mixed with 100 ml of orange juice (to mask GHB´s salty taste) and given orally at 03:30 after waking up the participants with an acoustic signal without completely turning on the light and while staying in bed. Afterwards, participants were allowed to immediately return to sleep. The median time required for the administration of the liquid drug (from lights-on at 03:30 to lights-off) was 3 min (range: 1–10 min), including an optional toilet break if requested. GHB administration in the middle of the night was chosen because of GHB’s short half-life and the potency to enhance SWS in the second half of the night, when homeostatic sleep pressure is reduced, and physiological sleep is typically superficial [12]. Conversely, a single administration of GHB prior to sleep onset would result in wakefulness promotion during the middle of the night, thereby reducing clinical feasibility. GHB was dosed according to body weight at 50 mg/kg (range 2400–5200 mg), based on previous investigations showing a favorable sleep-promoting profile of single administration of GHB at this dose [12].

Trazodone HCl or solid placebo (mannitol) were administered orally at 23:30 and consisted of two capsules matched in appearance and taste. Trazodone was dosed according to body weight at 1.5 mg/kg (range 72–156 mg), which is a usual dose for the hypnotic use of the drug, while the antidepressant dose lies at about 300 mg/day [13]. Trazodone was administered 30 min prior to bedtime, to allow for onset of its sleep-promoting effects (peak concentrations expected 1 h after administration) [13]. Although it differs from GHB in terms of its pharmacological profile and administration scheme, trazodone was selected as an active comparator due to its efficacy in promoting overall nocturnal SWS while causing limited daytime sleepiness in a clinical setting.

All study medications were provided by the pharmacy of the University Hospital of Psychiatry Zurich. Balanced, block-wise randomization was performed by an experienced pharmacist at the hospital pharmacy. Both solid and liquid placebos were matched to the appearance and taste of the respective medications to ensure double-blinding of the entire study team.

### Assessments

During experimental nights, PSG was recorded with dedicated amplifiers (SIENNA Ultimate, EMS Handels GmbH) according to the rules of the American Academy of Sleep Medicine (AASM). Recordings consisted of electroencephalography (EEG, 23 electrodes attached according to the international 10-20 system), bipolar electrooculogram, electromyogram, and electrocardiogram (see detailed description in Supplementary Methods). Visual sleep stage scoring observing the criteria of the AASM was done by two independent scorers using Rembrandt Analysis Manager (version 8, Embla Systems). Apparent discrepancies between scores were resolved by a third scoring expert. The following sleep variables were computed: (i) total sleep time (TST); (ii) sleep onset latency (SOL); (iii) wake-after-sleep-onset (WASO); (iv) duration of sleep stages (i.e., non-REM [NREM] stage 1 [N1], NREM stage 2 [N2], NREM stage 3 [N3, SWS], and REM sleep); and (v) sleep efficiency ([SE] = [TST/time-in-bed] × 100). The duration of sleep stages is reported as percentage of TST.

EEG power spectra were computed by a Fast-Fourier transform based on 4 s epochs (Hanning window, linear detrending, 50% overlap) using MATLAB (version 24.2, MathWorks Inc) and the EEGLAB toolbox. Spectra between 0.5 and 20 Hz of the EEG signal were investigated [12]. The average, all-night spectral power was computed across all epochs of a given sleep stage (N2, N3, NREM, REM). The evolution of spectral power within the slow-wave activity (SWA) range (0.5–4 Hz) and within the spindle frequency activity (SFA) range (11–16 Hz) was also specifically analyzed across consecutive NREM-REM sleep cycles. For this purpose, the sleep cycles according to Feinberg and Floyd criteria [14] were calculated in R using the SleepCycle algorithm [15]. For sleep spindle analysis, spindles were automatically detected using the python-based YASA algorithm (Version 3.12.6) [16]. NREM spindle density and amplitude were extracted separately for slow spindles (11–13.5 Hz) and fast spindles (13.5–16 Hz) [17] (see detailed description in Supplementary Methods).

Each participant’s morning sustained attention was assessed at 09:00 using a 10-min PVT. Reaction times (RT) below 100 ms were excluded as false starts. The median RT and the numbers of lapses (trials with RT > 500 ms) across the 10 min were analyzed as indications of sustained vigilant attention [18].

The N-back task was used to assess next-day working memory at 9:10. Participants were presented with a series of letters on a computer screen and asked to press a button if the current stimulus was presented 1, 2, or 3 steps back. Median speed (1/[RT]; including only correct answers with RT > 100 ms) and accuracy ([correct hits] + [correct rejections]/[total responses]) were calculated according to previous reports [19].

Plasma samples were collected at 8:30 using EDTA vacutainers, directly centrifuged at 2000 × *g* for 15 min, and then stored at −80°. BDNF concentrations were measured in a single batch at the end of the study using a commercial kit on a highly sensitive, automated, immunoassay platform (ELLA, Biotechne). BDNF was assessed because it has been suggested as a biological marker of rapid antidepressant response, also in the context of SWS modulation [20].

The Positive and Negative Affective Scale (PANAS) was used to assess momentary positive and negative affects before (22:30) and in the morning after (09:30) each experimental night (24:00–07:30). The Karolinska Sleepiness Scale (KSS) was used for the assessment of sleepiness directly before solid drug administration (23:30) and upon awakening (07.35). After awakening, a morning questionnaire (MQ, 08:00) was used to assess self-reported sleep quality at each experimental night.

### Outcomes

The primary and secondary outcomes have been preregistered at ClinicalTrials.gov (identifier: NCT04082806). For the current study, following primary outcomes were included based on their clinical relevance: nocturnal SWS (also called “deep sleep” or “stage N3 sleep”), the performance on the PVT (median RT and lapses) the performance on the N-back task (speed and accuracy), and plasma BDNF levels. The definition of PVT and N-back variables follows previous work from our and other labs [19, 21, 22]. Secondary outcomes were self-reported sleep quality (MQ), post-awakening sleepiness (KSS), and next-day affective state (PANAS). Exploratory outcomes included TST, WASO, and SOL, sleep stages N1, N2, REM, SE, EEG power spectra, and spindle analysis. The drug effects on the primary outcomes of memory consolidation (i.e., EPMT, PAWL, FSST, see ClinicalTrials.gov) and further electrophysiological variables will be published separately.

### Statistical analysis

All statistical analyses were computed with R-Studio (version 2023.9.1.494, R-Studio Core Team), and JASP. The significance level was set at p < 0.05 (two-tailed). Normal *Q*–*Q* and Tukey-Anscombe (residuals vs. fitted) plots were examined to assess model assumptions and goodness-of-fit. Variables with non-normal distributions were log- or power-transformed. To screen for drug effects on primary, secondary and exploratory outcomes, linear mixed-effects models (LMER) for continuous data and generalized linear mixed-effects models (GLM) of the Poisson family for count data were employed (R-package “lme4,” Version 1.1.34). These models incorporated all available data including from participants with incomplete datasets. Drug and experimental night order were included as within-subject factor, and subject ID as a random effect. Following model fitting, analyses of variance (ANOVA) were performed, to assess the overall significance of the fixed effects. If the condition was identified as significantly influencing an outcome variable, pairwise comparisons of estimated marginal means for each experimental condition were conducted using the “emmeans” package (Version 1.8.9). Therefore, the number of final comparisons considered was reduced to 10 variables and study-wise type 1 error strongly reduced. Considering the high degree of collinearity between variables and within subjects (e.g., many PSG variables are per definition strongly correlated), no correction for multiple comparison was performed on the post-hoc tests. An additional false discovery rate (FDR) analysis was performed to control for the total number of preregistered primary outcomes (n = 9). Spectral data were statistically analyzed with a linear mixed-effects model (R-package “nlme;” Version 3.1), while controlling for inter-frequency bin correlations, by an autoregressive moving average model and comparing bin-wise with general linear hypothesis tests and FDR correction. Standardized effect sizes (ES) are reported for post-hoc pairwise comparisons using the “eff_size” function of the “emmeans” package. Values of −0.8 < ES > 0.8 are considered to reflect large effects irrespective of the corresponding p-value. The initial sample size was selected according to power analysis showing that, given a power 1-β of 85%, α = 5%, and n = 30, medium effects (f = 0.21 equivalent to ES = 0.42) can be reliably detected (F-test for repeated measure ANOVA, within-subject effects, correlation among repeated measures = 0.65). A post-hoc calculation confirmed sufficient statistical power (1-β = 0.99, α = 5%) for the main outcome variable (ES for SWS = 2.03) despite the lower sample size (n = 23) than initially planned.

## Results

### Demographic and clinical characteristics

Sample characteristics are summarized in Table 1. All participants were under stable antidepressant medication and had a current depressive episode with severity level from mild to severe according to HAMD (score ≥ 8). The mean PSQI total score at baseline was above threshold (>5) for poor sleep quality [23].

**Table 1:** Sociodemographic and clinical characteristics at screening.

VARIABLES	MEAN	SD	RANGE
Age, years	26.8	6.5	20–45
Sex, f/m	16/7	-	-
BMI, kg/m^2^	23.1	3.4	18.2–29.4
Ethnicity (self-report), caucasian/asian/african/other	20/1/1/1	-	-
Education status, I/II/III/IV	0/1/15/7	-	-
AUDIT, score (alcohol)	3.6	3.3	0–15
CUDIT, score (cannabis)	1.7	2.3	0–7
FTND, score (nicotine)	1.7	0.5	1–2
PSQI time in bed, min	8.8	2.2	6–11
PSQI sleep latency, min	19.2	17.8	2–90
PSQI sleep efficiency, %	87.4	15.3	45.5–100
PSQI total, score	7.1	2.6	4–14
Medication, SSRI/SNRI/NDRI/vortioxetine/herbal	13/4/4/1/1		
MDD episodes, n	4.1	4.0	1–20
MDD duration, years since first symptoms	8.1	5.7	1–24
MADRS, score	18.7	6.2	8–31
HAMD, score	17.4	5.4	8–29
BDI, score	19.2	8.8	2–37
Hospitalizations, n	1.3	1.8	0–6

Table reports means ± standard deviations. Education status (highest degree): I = primary school, II = lower secondary level, III = upper secondary level, IV = tertiary level.

*BMI* Body Mass Index, *AUDIT* Alcohol Use Disorders Identification Test, *CUDIT* Cannabis Use Disorders Identification Test, *FTND* Fagerström Test for Nicotine Dependence, *PSQI* Pittsburgh Sleep Quality Index, *SSRI* Selective Serotonin Reuptake Inhibitors, *SNRI* Serotonin and Norepinephrine Reuptake Inhibitors, *NDRI* Norepinephrine and Dopamine Reuptake Inhibitors, *MDD* Major Depressive Disorder, *MADRS* Montgomery-Åsberg Depression Rating Scale, *HAMD* Hamilton Depression Rating Scale, *BDI* Beck Depression Inventory.

### Drug effects on sleep architecture: primary and explorative outcomes

**Fig. 2:**
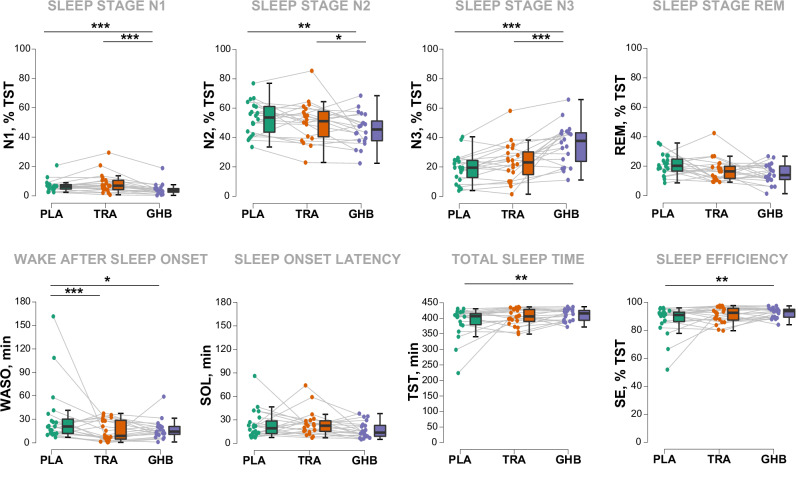
Drug effects on nocturnal sleep architecture. Boxplots and jittered raw data are visualized. The duration of the sleep stages N1, N2, slow-wave-sleep (SWS, N3) and rapid eye movement (REM) sleep are reported as % of total sleep time (TST). The duration of wake-after-sleep-onset (WASO), sleep onset latency (SOL), and TST are reported in minutes. Sleep efficiency (SE) is calculated as SE = (TST) / (total time-in-bed) × 100.

Drug effects on PSG variables are summarized in Fig. 2. Compared to placebo, GHB induced a strong elevation of SWS (% of TST: +15.8; 95% CI, +10.8, +20.8 p = <0.001; ES = 2.03), while shortening N1 (% of TST: −2.9; 95% CI, −4.6, −1.4; p < 0.001; ES = 2.01), N2 sleep (% of TST: −8.5; 95% CI, −13.9, −3.2; p = 0.003; ES = 1.03) and WASO (minutes: −19.5; 95% CI, −35.5, −3.6; p = 0.025; ES = 0.74). GHB also increased SE (% of TST: +5.5; 95% CI, +1.1, +9.9; p = 0.011; ES = 0.84) and TST (minutes: +24.0; 95% CI, +4.5, +43.4; p = 0.011; ES = 0.85) compared to placebo. Trazodone did not induce a significant prolongation of SWS (% of TST: +3.6; 95% CI, −1.4, +8.6; p = 0.151; ES = 0.46), but tended to elevate TST (minutes: +18.2; 95% CI, −1.1, +37.6; p = 0.087; ES = 0.56) and SE (% of time-in-bed: +4.3; 95% CI, −0.1, +8.7; p = 0.072; ES = 0.58), while reducing WASO (minutes: −21.5; 95% CI, −37.4, −5.6; p < 0.001; ES = 1.26). Compared to trazodone, GHB increased SWS (% of TST: +12.2; 95% CI, +7.3, +17.1; p < .001; ES = 1.57), while reducing N1 (% of TST: −4.5; 95% CI, −6.1, −2.9; p < 0.001; ES = 2.06) and N2 (% of TST: −6.3; 95% CI, −11.6, −1.0; p = 0.020; ES = 0.75) sleep. No differences between conditions were observed for SOL and the duration of REM sleep.

### Drug effects on quantitative sleep EEG and spindle activity: explorative outcomes

**Fig. 3:**
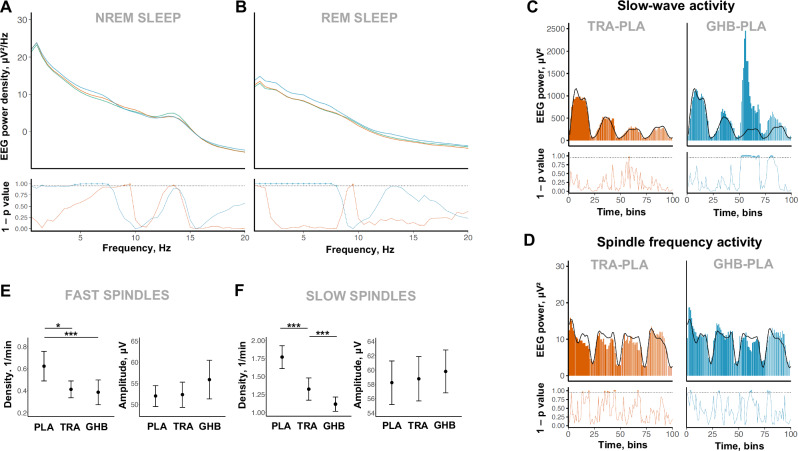
Drug effects on quantitative EEG variables. **A**, **B** All-night EEG spectral power density following placebo (green line), trazodone (red line), and GHB (blue line) administration. Mean values were plotted for NREM sleep (stages N2 and N3) and REM sleep. The significance values for the alternative hypothesis (1 − p value) are visualized in the y-axis at the bottom of the panels. Dots indicate frequency bins (0.5 Hz resolution), which differed significantly from placebo (p_FDR _< 0.05 or [1 – p_FDR_] > 0.95). **C**, **D** Time course of slow-wave activity (spectral power between 0.5 and 4.0 Hz; **C**) and spindle frequency activity (spectral power between 11–16 Hz; **D**) across consecutive NREM-REM sleep cycles after trazodone (red bars), GHB (blue bars) and placebo (black line) administration. Each NREM sleep episode was divided in 20 and each REM sleep episode in 5 temporal bins. The relative differences in duration are visualized by bar width. Dots at the bottom of the panels indicate the temporal bins, which differed significantly from placebo (p_FDR _< 0.05 or [1 –  p_FDR_] > 0.95). **E**, **F** Mean values and standard errors for the density and amplitude of fast (11–13.5 Hz; **E**) and slow (13.5–16 Hz; **F**) sleep spindles across the separate conditions.

**Fig. 4:**
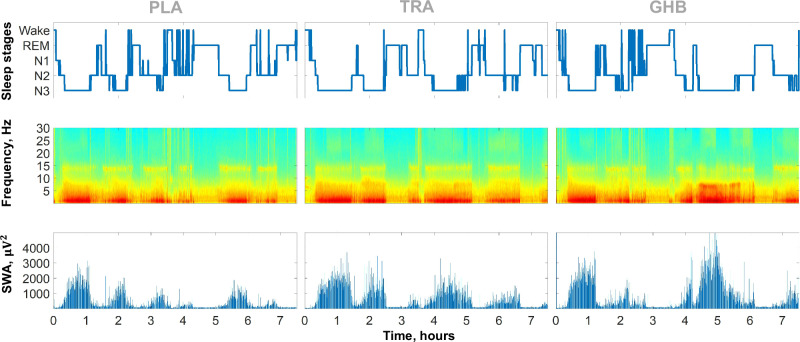
Hypnogram (top row), time frequency analysis (middle row), and time course of slow-wave activity (SWA) (bottom row; spectral power density within 0.5–4 Hz) of a single subject after intake of placebo (left panel), trazodone (middle panel), and GHB (right panel).

Drug effects on all-night EEG spectral power in NREM and REM sleep, on the evolution of SWA and SFA across consecutive sleep cycles, and on main spindle characteristics in NREM sleep are summarized in Fig. 3. Representative data from a single participant are visualized in Fig. 4. GHB increased EEG spectral power in the delta and theta bands in both NREM (frequency bins with p_FDR_ < 0.05: 1.5 Hz and 4.5–7.5 Hz) and REM sleep (frequency bins with p_FDR_ < 0.05: 0.5–8.0 Hz). Trazodone increased alpha spectral power in both NREM (frequency bins with p_FDR_ < 0.05: 9–9.5 Hz) and REM sleep (frequency bin with p_FDR_ < 0.05: 9.5 Hz) while it decreased spindle spectral power in NREM (frequency bin with p_FDR_ < 0.05: 13.5 Hz). Looking at sleep cycles separately, GHB strongly increased SWA and slightly decreased SFA in the third and fourth NREM sleep episodes, confirming the expected promotion of SWS in the second half of the night (all time bins with significant differences for GHB vs. placebo after FDR correction are highlighted in blue in Fig. 3C, D). Trazodone also slightly increased SWA in the third NREM sleep episode, while it decreased SFA in the first, second, and third NREM sleep episodes (all time bins with significant differences for trazodone vs. placebo after FDR correction are highlighted in red in Figs. 3C, D). Spindle analyses revealed that GHB decreases both the density of slow (1/min: −0.67; 95% CI, −0.44, −0.91; p < 0.001; ES = 1.87) and fast (1/min: −0.25; 95% CI, −0.09, −0.41; p < 0.001; ES = 1.32) spindles. Trazodone also decreased the density of both slow (1/min: −0.47; 95% CI, −0.24, −0.70; p < 0.001; ES = 1.37) and fast (1/min: −0.24; 95% CI, −0.08, −0.40; p = 0.040; ES = 0.69) sleep spindles in NREM sleep.

### Drug effects on next-day vigilance and working memory: primary outcomes

The statistical analyses of the PVT revealed no drug effects on median RT (LMER with Gaussian distribution) when corrected for experimental night order (Table 2). By contrast, significant drug effects were observed on the number of lapses (GLM with Poisson distribution), including the correction for experimental night order. The post-hoc analyses showed that after GHB, patients produced less lapses than after trazodone (ratio of means: −0.50; 95% CI, −0.21, −0.78; p < 0.001; ES = 0.50) and placebo (ratio of means: −0.51; 95% CI, −0.21, -0.80; p < 0.001; ES = 0.51). No difference was observed between the trazodone and placebo conditions (+0.36; 95% CI, −2.2, +2.9; p = 0.95; ES < 0.01). No drug effects were observed on n-back performance for both speed and accuracy when corrected for experimental night order (Table 2).

**Table 2:** Drug effects on sustained attention, working memory, self-reported sleep quality, post-awakening sleepiness, and affective state.

VARIABLES	PLACEBO MEAN ± SD	PLA-TRA P-VALUE	TRAZODONE MEAN ± SD	TRA-GHB P-VALUE	GHB MEAN ± SD	PLA-GHB P-VALUE	P-VALUE CONDITION
PVT, median RT	307.8 ± 44.2		320.3 ± 44.7		307.5 ± 33.8		0.16
PVT, lapses	5.5 ± 7.6	0.95	7.7 ± 11.8	**<0.001**	3.4 ± 3.6	**<0.001**	**<0.001**
N-back, median speed	1.8 ± 0.5		1.7 ± 0.5		1.8 ± 0.5		0.64
N-back, accuracy	0.87 ± 0.10		0.86 ± 0.11		0.88 ± 0.10		0.88
MQ awakening, n	2.1 ± 1.3		1.7 ± 1.4		1.6 ± 1.3		0.28
MQ time awake, min	26.4 ± 50.5		18.8 ± 29.8		12.7 ± 15.7		0.06
MQ superficial sleep, VAS	43.6 ± 16.1		45.8 ± 22.4		38.3 ± 18.7		0.38
MQ undisturbed sleep, VAS	48.8 ± 16.1		55.9 ± 18.7		53.0 ± 20.1		0.30
MQ awakening recovery, VAS	38.6 ± 18.2	0.059	29.2 ± 20.3	**0.019**	41.8 ± 25.8	0.64	**0.043**
MQ awakening energy, VAS	45.9 ± 12.5	0.086	39.6 ± 15.9	**0.003**	51.4 ± 17.0	0.20	**0.012**
KSS pre-sleep, score	6.2 ± 2.1		5.8 ± 1.8		5.6 ± 1.9		0.26
KSS post-awakening, score	6.1 ± 2.1	**0.028**	7.3 ± 1.6	**0.014**	5.9 ± 2.4	0.81	**0.025**
PANAS positive evening, score	22.3 ± 5.2		22.3 ± 6.0		21.8 ± 6.6		0.85
PANAS negative evening, score	16.0 ± 5.4		16.6 ± 5.7		15.1 ± 4.1		0.35
PANAS positive morning, score	24.0 ± 7.8		20.7 ± 8.0		23.5 ± 7.5		0.15
PANAS negative morning, score	14.5 ± 4.7		15.0 ± 3.9		14.6 ± 3.3		0.92

Table reports means ± standard deviations. Model statistics refers to LMERs with condition (placebo vs. trazodone vs. GHB) and night order as factors. Significance levels of posthoc pairwise comparisons between conditions are reported if the model was found significant for the variable condition.

*PVT* Psychomotor Vigilance Test, *RT* response time, *MQ* Morning Questionnaire, *VAS* Visual Analogue Scale (1–100), *KSS* Karolinska Sleepiness Scale, *PANAS* Positive and Negative Affective Scale.

*P*-values <0.05 are in bold.

### Drug effects on plasma levels of brain derived neurotrophic factor: primary outcome

Plasma BDNF levels did not differ among conditions when corrected for experimental night order (mean ± SD, pg/ml, for placebo vs. trazodone vs. GHB: 2544 ± 1180 vs. 2850 ± 1779 vs. 3222 ± 2663; p_CONDITION_ = 0.31). None of the primary outcomes did significantly change after FDR correction.

### Drug effects on self-reported sleep quality, post-awakening sleepiness, next-day affective state: secondary outcomes

Results of self-reported sleep quality (MQ), post-awakening sleepiness (KSS), and affective state (PANAS) across conditions are summarized in Table 2. Trazodone increased sleepiness upon awakening (KSS) compared to GHB (+1.4; 95% CI, +0.30, +2.5; p = 0.014; ES = 0.53) and placebo (+1.3; 95% CI, +0.15, +2.9; p = 0.028; ES = 0.69) conditions and reduced next-day energy level (−12.5; 95% CI, −20.5, −4.4; p = 0.003; ES = 0.94) and recovery feeling (−13.1; 95% CI, −23.9, −2.3; p = 0.019; ES = 0.74) compared to GHB. No drug effects on affective state (PANAS) were observed.

### Safety and tolerability

No serious adverse events occurred. In the GHB condition, 8 of 22 participants reported mild side effects, with nausea and dizziness being the most common. In the trazodone condition, 1 of 23 participants reported moderate and 7 of 23 mild side effects, with nausea and headache being the most common. No side effects were observed in the placebo group (0 of 22). No participant withdrew from the study because of adverse events. Notably, a predominance of side effects in female participants was observed for GHB (female vs. male, n = 8 of 15 vs. 0 of 7) but not for trazodone (female vs. male, n = 6 of 16 vs. 2 of 7). In the overall sample, there was no emergence of serious suicidal ideation as assessed by the suicidality screening form.

## Discussion

We demonstrated that GHB strongly increases SWS and reduces next morning attentional lapses in patients with MDD. GHB showed a more favorable sleep-promoting profile compared to trazodone, which currently represents one of the most often administered substances to treat disturbed sleep in MDD.

The observed effects of a single nocturnal administration of GHB on SWS are in line with our previous observation in healthy volunteers [12, 24]. In particular, we confirmed that GHB promotes EEG activity in delta/theta bands and strongly increases SWA in the two sleep cycles after administration. On the contrary, the reduction in REM sleep observed in a previous study was not confirmed in the current investigation [12]. These observations have relevant clinical implications because SWS has crucial roles in physiological brain processes (e.g., cellular homeostasis, synaptic plasticity, and removal of waste metabolites) and high-order brain functions (e.g., cognitive performance, memory consolidation, and affective state) [25]. The reduction of SWS in many MDD patients has been linked to affective symptoms, cognitive deficits, and neurodegenerative alterations [26]. In contrast, increased SWS has been associated with a reduction of depressive symptoms in naturalistic and experimental studies of MDD [27–29]. Therefore, SWS is considered a main clinical target to develop treatment interventions for MDD.

Among the current pharmacological options for the treatment of sleep disorders in psychiatry, most compounds either reduce SWS (e.g., benzodiazepines and z-drugs) [5] or increase daytime sleepiness (e.g., sleep-promoting antidepressants and second-generation antipsychotics) [30]. Thus, the observation of increased SWS while improving next-day vigilance represents a unique advantage of GHB over other current sleep medications. In the current study, GHB was associated with a reduction in PVT lapses, which are thought to be the result of perceptual, processing, or executive failures associated with reduced cognitive alertness. An increase in PVT lapses has been consistently associated with sleep deprived states and fatigue [22]. Thus, our results confirm that nocturnal GHB administration might promote next-day vigilance even after a single administration [31]. Here, GHB’s modulatory effects on the anterior cingulate cortex have been suggested as key mechanisms of its vigilance-enhancing properties [31, 32]. On the other hand, the lack of drug effects on the N-back task suggests that the vigilance-enhancing effects of a single GHB administration are not sufficient to produce a positive effect on working memory performance.

Despite inducing a strong prolongation of SWS and promoting next-day vigilance, GHB was not associated with an improvement of subjective sleep quality or next-day affective state. A similar discordance between subjective and objective measures of sleep have been frequently reported in people with insomnia [33]. Nonetheless, in consideration of the suggested causal link between SWS and depression, an improvement of affective state after SWS-promotion by GHB might have been expected. In previous neuroimaging studies, we demonstrated that a single nocturnal administration of GHB results in next-day neural adaptations (i.e., of functional brain networks and of glutamate metabolism), which could hint at rapid antidepressant properties [31, 32]. However, investigations on GHB in narcolepsy, Parkinson’s disease, and fibromyalgia suggest that repeated administration of GHB over 4–6 weeks are required to elicit favorable effects on symptom dimensions such as fatigue, cataplexy, and pain sensitivity [9–11]. Sleep-promoting antidepressants like trazodone and mirtazapine also require several days of treatment to show antidepressant properties [13]. In this context, the lack of effects of both drugs on plasma BDNF levels is not surprising, as recent evidence has challenged the robustness, and the biological and clinical utility of blood BDNF measures [34]. Future investigations including repeated administrations are therefore warranted to clarify potential effects of GHB on subjective sleep quality, affective state, and depressive symptoms. Moreover, the recent development of extended-release GHB formulations, which prolong the sleep-promoting effects of GHB over the entire night, might be particularly indicated for future studies [35].

Although the data are somewhat inconsistent, altered spindle physiology has been associated with MDD [36] and may mediate cognitive deficits in other psychiatric disorders [37]. Therefore, the GHB-induced reduction in SFA and spindle density might have an impact on sleep-related restorative processes. Nevertheless, SFA changes induced by GHB were less pronounced than SWA changes, and spindle density and amplitude after GHB didn’t differ from trazodone, whose long-term use has rather been associated with neuroprotection and cognitive enhancing effects [38]. Further investigation of the effects of GHB-induced spindle changes and spindle-slow wave coupling on memory consolidation and next-day cognitive performance is warranted.

Our study bears several limitations. The final sample size of n = 23 resulted in a lower statistical power than initially planned, thus, affecting the generalizability of our findings. Although the large effect size of the main outcome variables clearly provided sufficient statistical power, we cannot exclude type II statistical errors particularly for secondary and exploratory outcomes. The current investigation did not provide information on individual characteristics predicting treatment responses because of insufficient sample size to perform subgroup analysis. From a pharmacological perspective, the different administration regimens of GHB and trazodone hinder a direct comparison of their effects on electrophysiological sleep modulation. That said, the inclusion of trazodone provides evidence supporting the clinical efficacy of GHB not only in relation to placebo but also to one of the most commonly used medications in psychiatry. Even though GHB was overall well-tolerated, the higher prevalence of side effects in female participants might limit the applicability of GHB in a clinical context and clearly requires further investigations. Sex-dependent adaptations of the dose (lower dose or slower titration) might be necessary although larger studies in narcolepsy and fibromyalgia did not report similar sex-dependent differences in the tolerability. Finally, while the PVT and N-back tasks are well-established assessments of next-day vigilance and working memory, future studies should include a broader characterization of cognitive performance and additional measures of global functioning in daytime activities.

It is important to note, that GHB continues to be used in non-medical context, primarily for its euphoric and pro-sexual effects [39]. Here, GHB can induce pharmacological tolerance, and its regular use may carry the risk of developing a substance use disorder, particularly in vulnerable individuals [40]. However, the addictive potential of GHB (and its related effects on midbrain dopamine circuits) varies greatly with dose, with repeated low doses (recreational/non-medical use) paradoxically showing much higher addictive potential (and dopamine release) than higher doses (medical use) [41]. Coherently, the medical use of GHB in patients with narcolepsy with cataplexy is associated with a very low risk of addiction and has proven to be safe by a post-marketing surveillance system [42]. In any case, a cautious consideration of the risks and benefits of GHB use in psychiatry should await future evidence from trials conducted in the clinical context.

In conclusion, we provide evidence of favorable sleep-promoting effects of GHB in patients with MDD. This proof-of-concept study represents the first experimental validation of single GHB administration to promote sleep in a clinical psychiatric population. Overall, our findings support further investigation of GHB as a promising sleep medication candidate in patients with MDD and in psychiatry in general.

## Supplementary information

Supplemental Material

## Data Availability

Anonymized data will be shared by request with any qualified investigator with an institutional review board approval for the purposes of validation and/or replication using our center’s established procedures for sharing data.
