# Supplementary material for: Gamma-hydroxybutyrate to promote slow-wave sleep in major depressive disorder: a randomized crossover trial
Source: Neuropsychopharmacology. 2025 Apr 14;50(8):1237–44. doi: 10.1038/s41386-025-02104-4 (PMC12170893; doi:10.1038/s41386-025-02104-4)
Supplement: Supplementary file 1 — Supplemental Material [file 41386_2025_2104_MOESM1_ESM.docx]

**Gamma-hydroxybutyrate to promote slow-wave sleep in major depressive disorder: a randomized crossover trial**

Francesco Bavato, MD^1^, Laura K. Schnider^2^, Dario A. Dornbierer, PhD^1^, Julia R. Di Floriano^1^, Benjamin Stucky^2^, Nicole Friedli^1^, Marina Janki^1^, Boris B. Quednow, PhD^1,3^, Hans-Peter Landolt, PhD^2,3,4^, Oliver G. Bosch, MD^1^, Erich Seifritz, MD^1,3,4^

*^1^Department of Adult Psychiatry and Psychotherapy, University Hospital of Psychiatry Zurich, University of Zurich, Zurich, Switzerland*

*^2^Institute of Pharmacology and Toxicology, University of Zurich, Zurich, Switzerland*

*^3^Neuroscience Center Zurich, University of Zurich and Swiss Federal Institute of Technology Zurich, Zurich, Switzerland*

*^4^Sleep & Health Zurich, University of Zurich, Zurich, Switzerland*

**Supplementary Methods**

***Inclusion and exclusion criteria.*** All participants were assessed by an experienced study physician regarding their general health and psychiatric history. Inclusion criteria were as follows: diagnosis of major depressive disorder (MDD) according to the Diagnostic and Statistical Manual of Mental Disorders (DSM-5); stable antidepressant treatment (e.g., selective serotonin reuptake inhibitors [SSRI] or serotonin–norepinephrine reuptake inhibitors [SNRI]); age between 20-65 years; no or low dependence on nicotine according to Fagerström Test for Nicotine Dependence (total score <3).^1^ Exclusion criteria entailed: known hypersensitivity or allergy to trazodone; daily intake of contraindicated drugs according to the Swiss federal medical product information (e.g., barbiturates, opioids); intake of any sleep-promoting medication, such as benzodiazepines or z-drugs three days before an experimental night or on a regular basis; any axis-I DSM-5 psychiatric disorder other than MDD (e.g., schizophrenia, bipolar disorder); neurological disorders or head injury; any clinically relevant medical diseases; family history of axis-I psychiatric disorders other than depression and stress-related disorders (e.g., schizophrenia or bipolar disorder); any form of substance dependence or regular use of illegal substances (lifetime use per substance greater than five times, with exception of occasional cannabis use); lifetime history of GHB use; for female participants: pregnancy, breast feeding, planned pregnancy during the course of the study, or lack of safe contraception (self-reported); inability to follow the procedures of the study because of language problems (i.e., attendance of primary school in a German-speaking country required); participation in another study with investigational psychoactive substances within 30 days preceding and during the present study; dietary restrictions. Prior to definitive enrollment into the study, all participants underwent a polysomnography (PSG) examination to exclude sleep-related disorders such as sleep apnea, restless legs syndrome and sleep onset REM sleep. Additionally, participants were required to abstain from caffeine after 13:00 on experimental days and from alcohol for at least 24 hours prior to measurements. Urine samples were taken on each test night, to ensure abstinence from illegal substance use and to exclude pregnancy. Participants were instructed to keep a regular sleep-wake rhythm with seven to eight hours of time-in-bed from 23:00-24:00 to 07:00-08:00 during two weeks prior to the first experimental night and in the weeks between the experimental nights. To ensure compliance with this requirement, participants wore an actimeter on the non-dominant arm and kept a sleep-wake diary.

***Detailed clinical assessments:*** Screening night: Structured interviews for depressive symptoms were performed by an experienced study physician with at least 3-years’ experience in clinical psychiatry. Clinician administered questionnaires included the Montgomery-Åsberg Depression Rating Scale (MADRS) and the Hamilton Depression Rating Scale (HAMD). Self-reported sleep quality was assessed at the screening session with the Pittsburgh Sleep Quality Index (PSQI). Experimental nights: Self-reported depressive symptoms were assessed at the beginning of each session with the Beck Depression Inventory (BDI, 21:30). The Positive and Negative Affective Scale (PANAS) was used to assess momentary positive and negative affects before (22:30), and in the morning after (09:30) the experimental night (24:00-07:30). The Karolinska Sleepiness Scale (KSS) was used for the assessment of current sleepiness directly before solid drug administration (23:30) and upon awakening (at 07.35). An evening questionnaire (EQ, 23:30) was used to assess the intake of any caffeinated or alcoholic beverages, daytime naps, and medication intake. After awakening, the Morning Questionnaire (MQ, 08:00) was used to assess self-reported sleep quality at each experimental night. In the MQ, participants were asked to subjectively quantify time awake after lights-off (min), number of nocturnal awakenings (count) during the past night, and to rate on visual analogue scales (range 1-100) sleep-related items (sleep: “deep” vs. “superficial”; sleep: “quiet” vs. “restless”; current state: “recovered” vs. “tired”; current energy level: “full of energy” vs. “listless”). A suicide screening form (adapted from the suicide status form II) was performed at the end of each session to exclude acute suicidality.

***Polysomnography data acquisition.*** During experimental nights, PSG recordings according to the rules of the American Academy of Sleep Medicine (AASM)^2^ and consisting of electroencephalography (EEG), bipolar electrooculogram (EOG), electromyogram (EMG), and electrocardiogram (ECG) were performed. For the EEG, 23 electrodes were attached according to the international 10-20 system. Twenty electrodes were used for EEG recording (Fp1, Fp2, F3, F4, F7, F8, Fz, T3, T4, T5, T6, C3, C4, Cz, P3, P4, Pz, O1, O2, Oz), two as reference electrodes (A1, A2) and one as a ground electrode placed on the forehead. Placement of electrodes was determined by first measuring the head circumference and subsequently marking the appropriate points with the aid of a fitted cap (EasyCap). The EOG electrodes were placed diagonally across the eyes (E1, E2). Three electrodes were taped to the chin for recording the submental EMG (Chin1, Chin2, ChinZ). Two further EMG electrodes were vertically attached to the tibialis posterior of the right leg. All-night PSG was recorded with a dedicated PSG amplifier (SIENNA Ultimate, EMS Handels GmbH, Korneuburg, Austria). The signals were conditioned by a 50 Hz notch-filter to remove line-noise, a high-pass filter (EEG: 0.5 Hz; EMG: 5 Hz; ECG: 1 Hz) and an antialiasing low-pass filter (EEG, EMG: 100 Hz; ECG: 30 Hz), digitized and saved with a resolution of 512 Hz (sampling frequency of 512 Hz).

***Polysomnography data processing.*** Recordings were segmented into 30-second epochs and a sleep stage was determined for each epoch. Visual sleep stage scoring was done using Rembrandt® Analysis Manager (version 8; Embla Systems) according to the criteria of the AASM by two independent scorers. Discrepancies across scores were then resolved by a third scoring expert. For sleep stage scoring, EEG derivations F3, C3, and O1, as well as EOG E1 and E2 were referenced to linked mastoids. For chin muscle tone, either Chin1 or Chin2 referenced against the central chin electrode (ChinZ) were considered. Study participants could choose their bedtime (either at 23:00 or at 24:00) but to simplify descriptions and data presentations, we will only refer to the 24:00–07:30 rhythm. As the study medications were administered at two points during the night, with trazodone or solid placebo at 23:30 and GHB or liquid placebo at 03:30 (see Table 1), total time in bed (TIB) was considered as the sum of the nocturnal segments (from lights-off to lights-on) of the first (S1; 24:00-03:30) and second (S2; 03:30-07:30) night halves. The median time required for the administration of the liquid drug (from light on to light off at 03:30) was 3min (range: 1 to 10min) including an optional toilet break if requested. For analysis of sleep architecture, sleep variables were computed for the entire TIB. The following sleep variables were computed: (i) total sleep time (TST); (ii) sleep onset latency (SOL); (iii) wake after sleep onset (WASO); (iv) duration of sleep stages (i.e., non-REM [NREM] stage 1 [N1], NREM stage 2 [N2], NREM stage 3 [N3, SWS], and REM sleep); and (v) sleep efficiency ([SE] = [TST/TIB] × 100). The duration of sleep stages is reported as percentage of TST.

**Quantitative EEG analysis.** The preprocessing as well as power spectra extraction of Electroencephalographic (EEG) data was done using MATLAB (version 24.2, MathWorks Inc) and the EEGLAB toolbox (eeglab2023.1). The EEG data was downsampled to 256 Hz, a bandpass filter from 0.5 – 40 Hz was used, and data were detrended. We detected transient artifacts, such as those caused by large body movements, by identifying elevated spectral values across multiple frequencies with a moving median function with a hamming window of 2 seconds with no overlap. The moving median (240 second windows) plus or minus the median absolute deviation (MAD) multiplied by values of 2 to 3.5 in increments of 0.1 for all frequencies were calculated. Values exceeding these thresholds were identified and frequencies with more than 30% of thresholds surpassed were marked. Only timepoints having more than 20% of marked frequencies were deemed as outliers. Channels were manually inspected and labeled as fully bad or bad only on the first or second half of the recording. Those parts that were labeled as bad were interpolated with spherical interpolation in the EEGLAB toolbox. Electrical noise was filtered by applying a 50 Hz and 100 Hz notch filter. Additionally, some narrowband artifacts (multiples of 9.1 Hz) produced by the device were reduced by calculating the Fast Fourier Transform (FFT) and setting the amplitude of the affected frequencies to the 1 Hz moving median of the surrounding frequencies together with a corresponding phase correction. The inverse FFT was applied to revert the corrected complex signal the time domain. We removed artifacts due to the R-peak of a heart rate by a previously developed algorithm.^3,4^ To address other artefacts, like eye movements, a sequential artifact subspace reconstruction method was used with cutoff 30 and 8 minute windows with 50% overlap, as described in work by Somervail and colleagues.^5^

For quantitative EEG analysis, data were segmented into 30-second epochs, and only artifact-free data were included in the analysis. For each epoch, power spectral density (PSD) was estimated using Welch’s method with 4-second Hanning windows, 50% overlap, and a frequency resolution of 0.5 Hz. PSDs were calculated from the F3 electrode for frequencies ranging from 0.5 to 20 Hz, and power values were converted to decibels (10·log₁₀(μV²/Hz)). The F3 electrode was selected because of the frontal predominance of SWS.^6^

For statistical analysis, a Linear Mixed-Effects Model (LME) was fitted using the nlme package in R to evaluate the effects of condition, frequency, and their interaction on EEG power. The model included fixed effects for condition * frequency, a random intercept for subject ID (~1 | id), and an AR(1) correlation structure (corAR1) to account for within-subject correlations. An ANOVA was conducted on the fitted model to assess main effects and interactions. Post hoc pairwise comparisons were performed within each frequency bin using estimated marginal means (EMMs) calculated via the emmeans package, with fdr correction for multiple comparisons.

**Sleep cycles analysis.** Sleep cycles were calculated in R using the SleepCycle algorithm according to previous publications.^7^ This algorithm is largely based on the detection criteria by Feinberg and Floyd.^8^ Epochs scored according to AASM criteria were used as input for the algorithm. N2 was defined as the start of the first sleep cycle. If a detected sleep cycle comprised more than 240 epochs (i.e., 120 min) excluding wakefulness, the NREM period was split in two, where the last epoch of lightening of sleep marked the ending of the first period. The first 4 NREM-REM cycles (if less than 4 were available, as many cycles as available) were included in the analysis. Each sleep cycle was divided into 20 temporal bins for NREM sleep and 5 bins for REM sleep. The differential time extension of each cycle was also calculated and visualized in Figure 3 in terms of relative bar width. To investigate the time course of spectral power variations across cycles, spectral power was specifically analyzed in the slow-wave activity (SWA) band (0.5–4 Hz) of the F3 electrode, and the spindle frequency activity (SFA) band (11–16 Hz) of the C3 electrode during al N2, N3, NREM (N2 + N3), and REM sleep epochs. The F3 vs. C3 electrodes were selected according to the frontal vs. central predominance of SWA and SFA in the EEG.

For statistical analysis on sleep cycles, the LME included condition * bin as fixed effects to assess the effects of condition, temporal bin, and spectral power. The model included condition * bin as fixed effects to assess their interaction on mean absolute EEG, with a random intercept for subject ID (~1 | id) and an AR(1) correlation structure (corAR1) to account for within-subject correlations.

**Sleep spindle analysis.** Following filtering and cleaning of the data, sleep spindles were automatically detected using the “yasa.spindles_detect” function in the python-based YASA algorithm (Version 3.12.6), largely based on the algorithm by Lacourse et al, using Matlab’s built-in support for Python integration.^9^ Spindles were detected in C3 and F3 EEG channels from separate and artefact-free N2 and N3 sleep stages. The algorithm processes filtered EEG data to detect sleep spindles using multiple features. It applies a 300 ms sliding window with a 100 ms step size to calculate the moving root mean squared (RMS). Using the same window and step size, it computes a moving correlation between the broadband EEG signal (1-30 Hz) and the spindle-band filtered signal. Additionally, the relative spectral power of the spindle band compared to the total spectral power is estimated using Short-Time Fourier Transforms (STFT) with 2 s windows and a 200 ms overlap. Sleep spindles were detected if all the following criteria were met: the moving RMS exceeded the threshold of RMSmean + 1.5 × RMSSD, the moving correlation surpassed 0.65, and the relative spectral power in the spindle band met the predefined threshold of 0.2. The exact beginning and end of a spindle were detected where two out of three thresholds were crossed. Spindles closer than 500ms were considered the same spindle. For the analysis, fixed band-widths were used to detect slow spindles on F3 (11-13.5 Hz) and fast spindles on C3 (13.5-16 Hz), matching their frontal, respectively central, predominance in the EEG.^10^ Total spindle count, NREM (N2/N3) spindle density, spindle duration and amplitude were extracted from the resulting data frame for further analyses.

**Supplementary Figures**


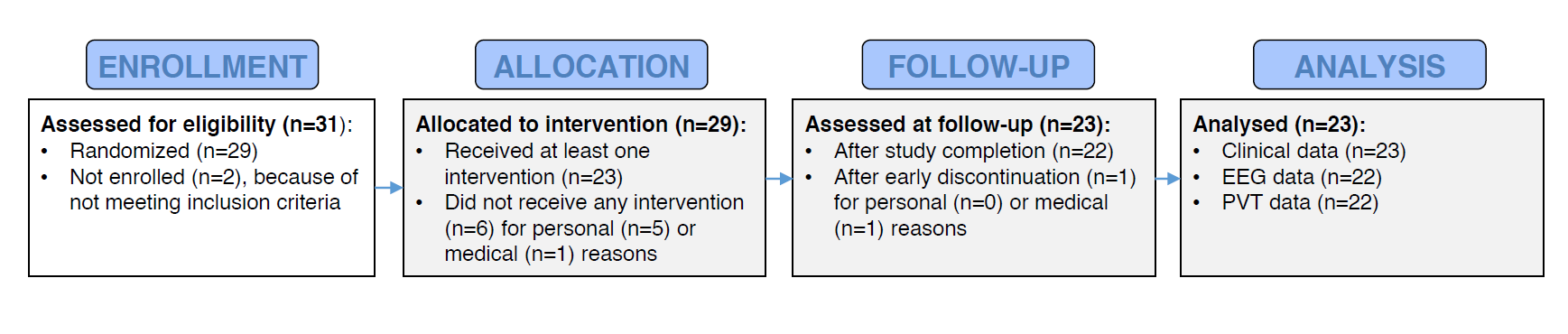


**Figure S1.** Study protocol diagram according to the CONSORT guidelines. Out of 31 patients assessed for eligibility, 29 patients were enrolled, 23 received at least one intervention and were considered for data analysis, and 22 patients completed the study. Six patients discontinued the study after enrollment but before the first experimental night because of personal (i.e., feeling anxious/unwell in the sleep lab environment, n=2; study protocol being too time consuming, n=2) or medical reasons (i.e., occurrence of new medical conditions, n=2). No participant withdrew from the study because of adverse events. One participant reported use of amphetamines and consequential clinical worsening a few days after the first experimental night (trazodone) and decided to withdraw from the study.

**References**

1 Heatherton, T. F., Kozlowski, L. T., Frecker, R. C. & FAGERSTROM, K. O. The Fagerström test for nicotine dependence: a revision of the Fagerstrom Tolerance Questionnaire. *British journal of addiction* **86**, 1119-1127 (1991).

2 Berry, R. B. *et al.* AASM Scoring Manual Updates for 2017 (Version 2.4). *J Clin Sleep Med* **13**, 665-666, doi:10.5664/jcsm.6576 (2017).

3 Skorucak, J. *et al.* Automatic detection of microsleep episodes with feature-based machine learning. *Sleep* **43**, doi:10.1093/sleep/zsz225 (2020).

4 Purcell, S. M. *et al.* Characterizing sleep spindles in 11,630 individuals from the National Sleep Research Resource. *Nature Communications* **8**, 15930, doi:10.1038/ncomms15930 (2017).

5 Somervail, R., Cataldi, J., Stephan, A. M., Siclari, F. & Iannetti, G. D. Dusk2Dawn: an EEGLAB plugin for automatic cleaning of whole-night sleep electroencephalogram using Artifact Subspace Reconstruction. *Sleep* **46**, zsad208 (2023).

6 Dijk, D. J. Regulation and functional correlates of slow wave sleep. *J Clin Sleep Med* **5**, S6-15 (2009).

7 Blume, C. & Cajochen, C. 'SleepCycles' package for R - A free software tool for the detection of sleep cycles from sleep staging. *MethodsX* **8**, 101318, doi:10.1016/j.mex.2021.101318 (2021).

8 Feinberg, I. & Floyd, T. C. Systematic trends across the night in human sleep cycles. *Psychophysiology* **16**, 283-291, doi:10.1111/j.1469-8986.1979.tb02991.x (1979).

9 Lacourse, K., Delfrate, J., Beaudry, J., Peppard, P. & Warby, S. C. A sleep spindle detection algorithm that emulates human expert spindle scoring. *Journal of Neuroscience Methods* **316**, 3-11, doi:<https://doi.org/10.1016/j.jneumeth.2018.08.014> (2019).

10 Fernandez, L. M. & Lüthi, A. Sleep spindles: mechanisms and functions. *Physiological reviews* (2020).

**Supplementary Tables**

| ***Supplementary Table 1. Drug effects on nocturnal sleep architecture*** | | | | | | | |
| --- | --- | --- | --- | --- | --- | --- | --- |
| **VARIABLES** | **PLACEBO**  **MEAN ± SD** | **PLA-TRA**  **P-VALUE** | **TRAZODONE**  **MEAN ± SD** | **TRA-GHB**  **P-VALUE** | **GHB**  **MEAN ± SD** | **PLA-GHB**  **P-VALUE** | **P-VALUE**  **CONDITION** |
| Total sleep time (TST), min | 383.8±55.1 | 0.087 | 404.4±26.5 | 0.36 | 408.3±19.7 | **0.011** | **0.026** |
| Sleep onset latency (SOL), min | 23.1±18.7 |  | 25.0±16.2 |  | 18.0±12.0 |  | 0.095 |
| Wake after sleep onset (WASO), min | 38.1±43.8 | **<0.001** | 15.2±13.5 | 0.10 | 18.4±14.7 | **0.025** | **0.001** |
| Sleep stage 1 (N1), % TST | 7.1±4.0 | 0.87 | 8.6±6.6 | **<0.001** | 4.3±3.8 | **<0.001** | **<0.001** |
| Sleep stage 2 (N2), % TST | 52.0±11.4 | 0.39 | 50.5±13.3 | **0.020** | 43.7±12.0 | **0.003** | **0.007** |
| Sleep stage 3 (N3 or SWS), % TST | 19.3±9.6 | 0.15 | 22.9±12.8 | **<0.001** | 35.3±13.6 | **<0.001** | **<0.001** |
| Sleep stage REM, % TST | 21.7±7.1 |  | 18.0±8.4 |  | 16.7±9.0 |  | 0.18 |
| Sleep efficiency (SE), TST/TIB | 86.3±12.1 | 0.072 | 91.0±5.5 | 0.41 | 91.9±3.9 | **0.011** | **0.024** |
|  |  |  |  |  |  |  |  |
| *Table reports means ± standard deviations. Model statistics refers to LMERs or GLMs in case of count data, condition (placebo vs. trazodone vs. GHB) and night order as factors. Significance levels of posthoc pairwise comparisons between conditions are reported if the model was found significant for the variable condition. Abbreviations: REM: rapid eye movement sleep; TIB: total time in bed; PVT: Psychomotor Vigilance Test; RT: response time.* | | | | | | | |

| ***Supplementary Table 2. Adverse events*** | | | |
| --- | --- | --- | --- |
| **VARIABLES** | **PLACEBO (n=22)** | **TRAZODONE**  **(n=23)** | **GHB**  **(n=22)** |
| Nausea (%) | 0 (0%) | 4 (17.4%) | 6 (27.3%) |
| Dizziness (%) | 0 (0%) | 1 (4.3%) | 2 (9.1%) |
| Headache (%) | 0 (0%) | 3 (13.0%) | 0 (0%) |
| Imbalance (%) | 0 (0%) | 1 (4.3%) | 0 (0%) |
| Excessive sleepiness (%) | 0 (0%) | 1 (4.3%) | 0 (0%) |
| Vomiting (%) | 0 (0%) | 1 (4.3%) | 0 (0%) |
| Nocturnal enuresis (%) | 0 (0%) | 0 (0%) | 1 (4.5%) |
| Abdominal pain (%) | 0 (0%) | 0 (0%) | 1 (4.5%) |
|  |  |  |  |
| *Numbers indicate number of participants reporting the events* | | | |
